# Secondary cytoreductive surgery for recurrent epithelial ovarian carcinoma: proposal for patients selection

**DOI:** 10.1038/sj.bjc.6602466

**Published:** 2005-03-15

**Authors:** T Onda, H Yoshikawa, T Yasugi, M Yamada, K Matsumoto, Y Taketani

**Affiliations:** 1 Department of Obstetrics and Gynecology, Faculty of Medicine, University of Tokyo, 7-3-1 Hongo, Bunkyo-ku, Tokyo 113-8655, Japan; 2 Department of Obstetrics and Gynecology, University of Tsukuba, 1-1-1 Tennoudai, Tsukuba, Ibaraki 305-8575, Japan

**Keywords:** ovarian cancer, recurrence, secondary cytoreductive surgery, prognosis

## Abstract

The value of secondary cytoreductive surgery (SCS) for recurrent ovarian cancer is still controversial. The aim of this study was to clarify candidates for SCS. Between January 1987 and September 2000, we performed SCS in 44 patients with recurrent ovarian cancer, according to our selection criteria, disease-free interval (DFI) >6 months, performance status <3, no apparent multiple diseases, age <75years and no progressive disease during preoperative chemotherapy, if undertaken. The variables were investigated by univariate and multivariate analyses. Of 44 patients, 26 (59.1%) achieved complete removal of all visible tumours at SCS. Secondary cytoreductive surgery outcome, complete or incomplete resection, was significantly related to overall survival (*P*=0.0019). As for variables determined before SCS, DFI >12 months, no liver metastasis, solitary tumour and tumour size <6 cm were independently associated with favourable overall survival after recurrence in the multivariate analysis. Patients with three or all four variables (*n*=31) had significantly better survival compared with the other patients (*n*=13) (47 *vs* 20 months in median survival, *P*<0.0001). In these patients, fairly good median survival (40 months) was obtained even in patients with incomplete resection. Secondary cytoreductive surgery had a large impact on survival of patients with recurrent ovarian cancer when they had three or all of the above-mentioned four factors at recurrence. These patients should be considered as ideal candidates for SCS.

Since Griffiths (Griffiths, 1975) first demonstrated the inverse relationship between residual tumour size after primary debulking and survival of ovarian cancer patients in 1975, many investigators have reproduced and confirmed this observation (Hacker *et al*, 1983; Vogl *et al*, 1983; Delgado *et al*, 1984; Conte *et al*, 1985; Louie *et al*, 1986; Neijt *et al*, 1987; Hainsworth *et al*, 1988; Sutton *et al*, 1989). Thus, the value of debulking of large tumour masses in the primary surgery of ovarian cancer has been generally accepted, and primary cytoreductive surgery followed by chemotherapy is considered to be a standard treatment procedure for patients with advanced ovarian cancer.

The cytoreduction contributes to removal of the tumour burden and relief of symptoms caused by tumours or massive ascites. In addition, the cytoreduction has another important effect on the sensitivity to postsurgical chemotherapy. By removing bulky tumours, the decreased growth fractions should increase (Norton and Simon, 1977) and poorly perfused anoxic cells should decrease. By reducing the number of cancer cells, the chance for cancer cells to undergo spontaneous mutations resulting in drug resistance should decrease (Goldie and Coldman, 1979). All these effects are believed to enhance the sensitivity to chemotherapy.

Theoretically, the favourable effects of cytoreduction may also be expected in patients with recurrent ovarian cancer. Recently, several investigators have reported the significant value of secondary cytoreductive surgery (SCS) in a subset of patients with recurrent ovarian cancer (Jänicke *et al*, 1992; Eisenkop *et al*, 1995, 2000; Vaccarello *et al*, 1995; Cormio *et al*, 1999; Zang *et al*, 2000, 2004; Munkarah *et al*, 2001; Scarabelli *et al*, 2001; Tay *et al*, 2002). The value of complete resection at the time of SCS for highly selected patients is in consensus in these recent reports. They reported a considerable number of factors related to good prognosis including longer disease-free interval (DFI), smaller size of residual tumour at primary cytoreductive surgery, good response to first-line chemotherapy, younger age at recurrence and smaller size of maximum tumour at recurrence. However, there is limited information regarding the ideal candidates for SCS. Although only preoperative or intraoperative variables before starting SCS should be analysed for selection of the candidate, these variables have been analysed together with SCS outcome in most previous studies. In addition, the follow-up periods of living patients were rather short (the median or average follow-up periods were between 1 and 4 years) (Jänicke *et al*, 1992; Vaccarello *et al*, 1995; Cormio *et al*, 1999; Zang *et al*, 2000, 2004; Munkarah *et al*, 2001; Scarabelli *et al*, 2001) in most of the previous reports.

Since 1987, we have performed SCS according to our criteria of patient selection in 44 out of 70 ovarian cancer patients who had recurrence after DFI. In the present study, the median follow-up period of living patients is 60 months after the initiation of treatment, SCS or chemotherapy before SCS, for recurrence. Using univariate and multivariate analyses of variables before starting SCS, we planned to clarify the ideal candidates for SCS among patients with recurrent ovarian cancer.

## PATIENTS AND METHODS

### Patient selection

Between January 1984 and December 1999, we treated 236 patients with stage I to IV epithelial ovarian cancer at the Department of Obstetrics and Gynecology, University of Tokyo Hospital. Our standard surgical procedures for ovarian cancer consist of total abdominal hysterectomy, bilateral salpingo-oophorectomy, infracolic or total omentectomy, and in advanced cases, debulking of tumour masses with maximum efforts. Patients with no or small intraperitoneal residual tumours (less than 2 cm in diameter) also underwent systematic retroperitoneal lymphadenectomy. The extent of retroperitoneal lymphadenectomy is pelvic lymph nodes only (1984–1986) or both pelvic and aortic lymph nodes (1987–1999). All but stage Ia patients underwent at least six cycles of cisplatin-based chemotherapies following surgery as described previously (Onda *et al*, 1998). Of the 236 patients, 204 (86%) achieved complete clinical remission after primary treatment.

By September 2000, 70 of the 204 (34%) patients had recurrence and, from January 1987 to September 2000, 44 of the 70 (63%) patients underwent SCS prior to or following chemotherapy. Administration of chemotherapy before SCS was decided based on various clinical factors including short DFI (DFI <12 months) and poor performance status (PS 3) defined by ECOG (Eastern Cooperative Oncology Group). Our selection criteria for SCS were as follows: (1) DFI >6 months, (2) age at recurrence <75 years, (3) PS 0–2 just before the surgery, (4) absence of apparent extensive intraperitoneal dissemination or multiple distant metastases and (5) no progressive disease during presurgical chemotherapy, if undertaken. There were three exceptions to the above-mentioned criteria for SCS. One patient with DFI <6 months (5 months) underwent SCS, because the recurrent site was expected to be limited to a solitary aortic lymph node by CT. The other two patients had PS 3 at surgery. One patient with three metastatic brain tumours underwent emergent brain surgery followed by *γ*-knife radiosurgery to one residual tumour (Kawana *et al*, 1997), and one patient underwent ileocaecal resection because of acute bowel obstruction. Before the treatment, informed consent was obtained from all of the patients.

### Chemotherapy

Of 44 patients, 21 (47.7%) received chemotherapy before SCS and all of 44 patients were treated with chemotherapy after SCS. In all, one to eight (median: 2) cycles of presurgical chemotherapy were performed in eight of 13 (61.5%) patients with DFI <12 months and 13 of 31 (41.9%) patients with DFI >12 months. In total, 44 patients received two to nine (median: 4) cycles of postsurgical chemotherapy.

In all, two to four cycles of presurgical chemotherapy were generally administered until beneficial response (partial or minor response) was observed. In two patients, second-line chemotherapy showed no beneficial response, and SCS was performed after successful third-line chemotherapy (seven and eight cycles in total). One patient received only a cycle of presurgical chemotherapy, because SCS could not be scheduled immediately after diagnosis of recurrence.

The number of postsurgical chemotherapy given was determined by SCS outcome and response to chemotherapy, evaluated by CT scan and serum level of CA125. Generally, three to four cycles of chemotherapy were planned for patients with no residual tumour and five to six cycles of chemotherapy were planned for patients with any residual disease. In principle, we gave at least two cycles of chemotherapy after the serum level of CA125 was normalised. Thus, three patients were treated with more than six cycles of chemotherapy after SCS. On the contrary, chemotherapy was discontinued before accomplishment of the planned cycles in five patients because rapid disease progression or severe adverse effects were observed during the planned cycles.

In presurgical and postsurgical chemotherapies, a platinum-based combination, CAP, EP or TJ, was used. The CAP regimen consisted of 600 mg m^−2^ of cyclophosphamide, 30 mg m^−2^ of doxorubicin and 50–75 mg m^−2^ of cisplatin. The EP regimen consisted of 80 mg m^−2^ of etoposide during days 1–5 and 75 mg m^−2^ of cisplatin. Paclitaxel was introduced in Japan in 1998 and, thereafter, a TJ regimen consisting of paclitaxel (175 mg m^−2^ over 3-h infusion) and AUC 5 of carboplatin was used as second-line chemotherapy.

### Statistical methods

Survival was measured from the day of starting treatment for recurrence, that is, the day of starting presurgical chemotherapy or the day of performing SCS. The survival curves were determined by the Kaplan–Meier product limit method (Kaplan and Meier, 1958). Factors influencing survival were analysed using the log-rank test (univariate) and Cox's proportional-hazards regression analysis (multivariate). These analyses were performed using a JMP program (SAS Institute Inc., USA). Contingency table analysis was performed using the *χ*^2^ test or *χ*^2^ test for trend.

## RESULTS

### Patient characteristics

The number of patients was three in stage I, two in stage II, 36 in stage III and three in stage IV according to the International Federation of Gynecology and Obstetrics (FIGO). Histology was serous type in 35, clear-cell type in three, endometrioid type in three, transitional cell type in two and mixed epithelial type in one. Median DFI was 18.5 months with a range of 5–58 months: one patient (2.3%) had 5 months, 12 (27.3%) had 6–12 months and 31 (70.5%) had >12 months. Median age at recurrence was 52 years with a range of 37–74 years. Median follow-up period of patients, excluding those who died, was 60 months with a range of 17–199 months from the initiation of treatment for recurrence.

### Surgery

Our attempt to perform SCS resulted in exploratory laparotomy in four patients (9.1%) due to the presence of unexpected extensive peritoneal tumours. Various debulking surgeries classified into four categories such as (1) gastrointestinal resection, (2) resection of other organs, (3) lymph node dissection and (4) other tumour debulking was performed with maximum efforts in the remaining 40 patients (90.9%). Among these patients, gastrointestinal resection (category 1) was required in 11 patients (25.0%), large bowel resection in nine patients (20.5%), small bowel resection in three patients (6.8%), partial gastrectomy in one patient and ileocaecal resection in one patient (2.3%), and one of the patients (2.3%) underwent sigmoid colostomy. Three patients had category 1 surgeries at two sites. Resection of other organs (category 2) was required in six patients (13.6%), splenectomy in three patients (6.8%), distal pancreatectomy in two patients (4.5%), partial liver resection in one patient, hysterectomy in one patient and brain tumour resection in one patient (2.3%). Two patients had category 2 surgeries at two sites. Regional or distant lymph node dissection (category 3) was performed in 12 patients (27.3%). Five patients (11.4%) underwent systematic aortic lymphadenectomy and one (2.3%) underwent both systematic pelvic and aortic lymphadenectomies. Selective dissections of the following lymph nodes were performed in six patients: aortic nodes in one patient, pelvic nodes in one patient, axillary nodes in one patient, portal nodes in one patient, inguinal nodes in one patient and mesenteric nodes in one patient (2.3%). Other tumour debulking (category 4) including removal of tumours in the remnant omentum, the diaphragmatic muscles and vaginal stump, and tumours on the visceral or parietal peritoneum including the under surface of the diaphragm, was performed in 22 patients (50.0%); omentectomy in seven patients; partial full-thickness diaphragm resection in one patient; resection of tumours around the vaginal stump in four patients (9.1%); peritoneum resection of disseminated tumours on the under surface of the diaphragm; and other peritoneal surfaces in 16 patients (36.4%). Six patients were counted twice because they underwent two types of category 4 surgeries. In all, 10 patients underwent two or three out of the above four categories of debulking surgery. No patients died within a month following SCS.

### Cytoreductive outcome and survival of patients

**Figure 1:**
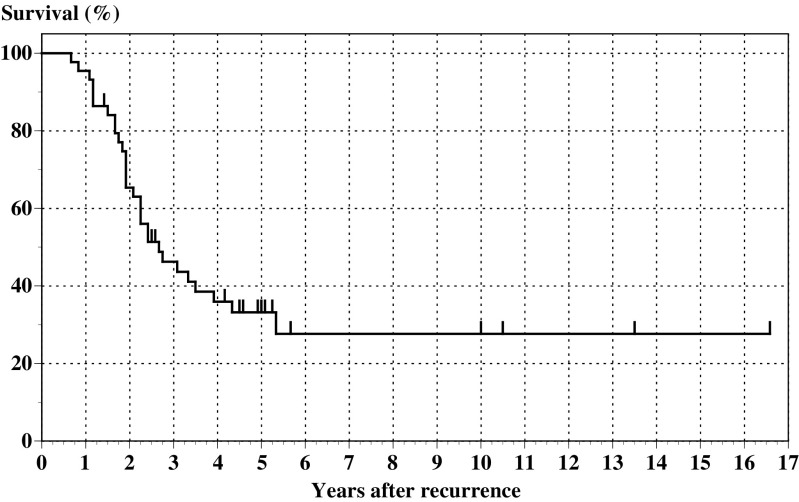
Survival of all 44 patients who underwent SCS.

**Figure 2:**
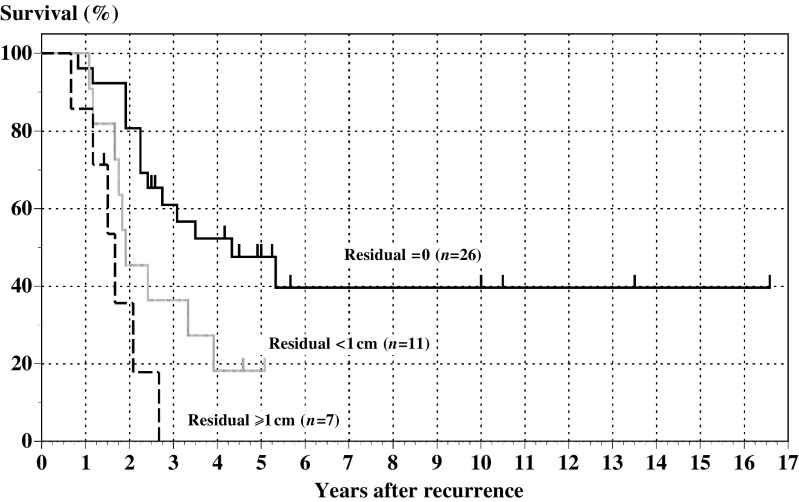
Outcome of SCS and survival. Survival of the patients with largest residual tumours 0, <1 and ⩾1 cm is shown in solid black, solid grey and dotted black line, respectively. The difference of survival is statistically significant (*P*=0.0007, log rank). There is no statistical difference in survival between patients with residual tumours <1 and ⩾1 cm (*P*=0.1314, log rank).

Among a total of 44 patients, complete resection of visible tumours was achieved in 26 patients (59.1%), largest residual tumours <1 cm in diameter were left in 11 patients (25.0%) and largest residual tumours ⩾1 cm in diameter were left in seven patients (15.9%). The median survival and 5-year survival of all patients who underwent cytoreductive surgery were 32 months and 33.2% (Figure 1), whereas the median survival and 5-year survival of 26 patients who had recurrence after complete remission achieved by primary treatment and did not undergo the surgery were 11 months and 3.9%. Figure 2 shows the survival of patients after the initiation of treatment for recurrence according to the outcome of SCS (SCS outcome). The median survival and 5-year survival after recurrence of the patients with largest residual tumours 0, <1 and ⩾1 cm were 52 months and 47.6%, 23 months and 18.2% and 20 months and 0%, respectively (*P*=0.0007, log rank). The overall survival of patients with no residual tumour was much better than that of patients with residual tumours (22 months in median survival and 12.0% in 5-year survival, figure not shown) with statistical significance (*P*=0.0019). There was no statistical difference in overall survival between patients with residual tumours <1 and ⩾1 cm (*P*=0.1314).

### Factors influencing survival in univariate analyses

Factors influencing overall survival after recurrence were analysed using univariate analyses. Factors analysed and the results of univariate analyses are listed in Tables 1 and 2. As for prognostic factors determined during primary therapy, univariate analyses revealed that peritoneal tumour spread (*P*=0.039), FIGO stage (*P*=0.045) and aortic lymph node metastasis (*P*=0.009) were significantly associated with overall survival after recurrence. Regarding prognostic factors determined at recurrence, univariate analyses revealed that DFI (*P*=0.002), presence of liver metastasis (*P*=0.005), number of recurrent tumours (*P*=0.007), size of maximum tumour (*P*<0.001) and SCS outcome (*P*=0.002) had significant associations with overall survival after recurrence.

### Factors influencing survival in multivariate analysis

To determine patient selection for the surgery, we performed multivariate analysis using statistically significant prognostic factors in univariate analyses. Out of eight significant factors, SCS outcome was omitted in the multivariate analysis because SCS outcome is not yet known on considering indications for the surgery, although SCS outcome had a statistically significant correlation with the number of recurrent tumours (*P*<0.001, *χ*^2^ test). The multivariate analysis using the remaining seven factors revealed that four factors determined at recurrence, specifically DFI, presence of liver metastasis, number of recurrent tumour and size of maximum tumour, were independently and significantly associated with survival after recurrence (Table 3). Additionally, the multivariate analysis using only these four factors confirmed that all four factors were independently and significantly associated with survival after recurrence. The relative risk (95% confidence interval) was 0.37 (0.20–0.68) for DFI >12 months, 0.23 (0.10–0.65) for absence of liver metastasis, 0.26 (0.12–0.48) for a solitary tumour and 0.20 (0.09–0.42) for size of maximum tumour <6 cm.

### Grouping of patients determined by the number of favourable prognostic factors

**Figure 3:**
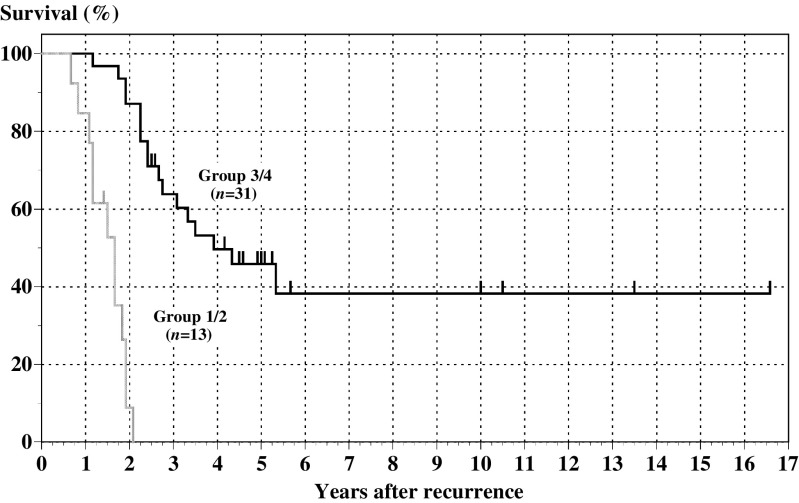
Comparison in survival between patients having one or two favourable prognostic factors (Group 1/2) and three or four favourable factors (Group 3/4). Survival of patients in Group 3/4 and Group 1/2 is shown as a solid black or solid grey line, respectively. Patients in Group 3/4 had significantly better survival compared with patients in Group 1/2 (*P*<0.001, log rank).

According to the number of favourable statuses among the above-mentioned four prognostic factors, that is, DFI >12 months, no liver metastasis, solitary tumour and tumour size <6 cm, patients were divided into four groups as follows: patients with all four favourable factors (Group 4, *n*=10), patients with three favourable factors (Group 3, *n*=21), patients with two favourable factors (Group 2, *n*=11) and patients with only one favourable factor (Group 1, *n*=2). There were no patients with zero favourable factors. Complete resection of visible tumours was achieved in 100% (10 of 10), 62% (13 of 21), 18% (two of 11) and 50% (one of two) of patients in Group 4, Group 3, Group 2 and Group 1, respectively. Apparently, a higher rate of complete surgical resection was achieved in patients with a larger number of favourable factors, and the distribution was statistically significant by contingency table analysis (*P*<0.001, *χ*^2^ test for trend). The 5-year survival of Group 4 was 88.9% and median survival was not reached. The 5-year survivals and median survivals of Group 3, Group 2 and Group 1 were 26.0, 0 and 0%, and 37, 20 and 10 months, respectively (figure not shown). The differences of overall survival were also statistically significant among the four groups (*P*<0.001, log rank) and between them (e.g. *P*<0.007 in Group 1 *vs* Group 2, *P*<0.001 in Group 2 *vs* Group 3 and *P*<0.001 in Group 3 *vs* Group 4, log rank). Figure 3 shows the combined survival of Group 4 and Group 3 and that of Group 2 and Group 1. Patients with three or all four favourable factors (Group 3/4) (*n*=31) had significantly better survival compared with those with less than three favourable factors (Group 1/2) (*n*=13) (median and 5-year survival; 47 months and 45.9% *vs* 20 months and 0%, *P*<0.001).

### Survival of patients determined by the number of favourable prognostic factors and SCS outcome

**Figure 4:**
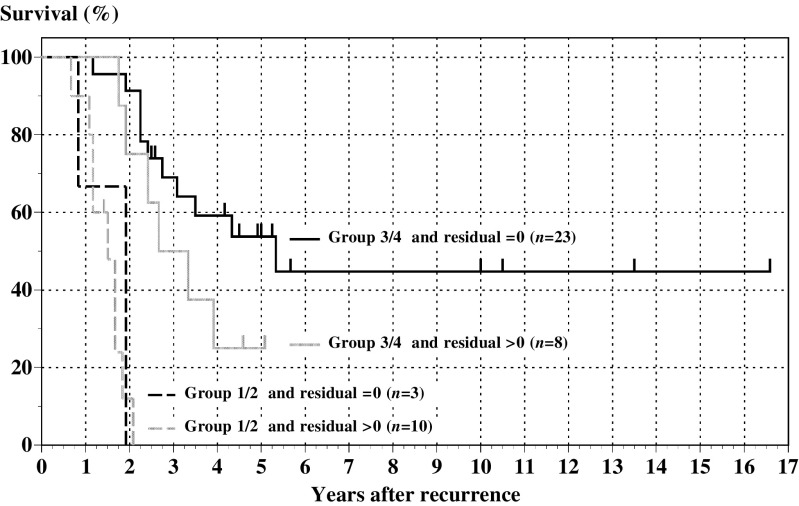
Survival in relation to SCS outcome and number of favourable prognostic factors. Survival of patients in Group 3/4 are shown as solid lines. Solid black line and solid grey line show the survival of patients with no residual tumour and residual tumour at SCS, respectively. Survival of patients in Group 1/2 are shown as dotted lines. Dotted black line and dotted grey line show the survival of patients with no residual tumour and any residual tumour at SCS, respectively.

Patients with three or all four favourable prognostic factors (Group 3/4) had better survival when complete surgical resection was achieved at the time of SCS (*n*=23) (64 months in median survival, 53.8% in 5-year survival). However, even when SCS left residual tumours, survival of the Group 3/4 patients (*n*=8) was fairly good (40 months in median survival, 25% in 5-year survival). On the other hand, Group 1/2 patients had poorer survival both in completely resected cases (*n*=3) and in incompletely resected cases (*n*=10) (23 and 18 months in median survival, and 0 and 0% in 5-year survival) (Figure 4).

## DISCUSSION

We achieved surgical removal of all visible tumours in 59.1% of patients at the time of SCS. Residual tumours <1 or ⩾1 cm in diameter were present in 25.0 and 15.9%, respectively. In line with previous reports, removal of all visible tumours at SCS contributed to long-term survival (Figure 2). The rate of complete resection (59.1%) in our series was a little lower than the rates reported by Eisenkop *et al* (2000), Landoni *et al* (1998) and Cormio *et al* (1999). However, in Landoni's study, the subjects were restricted to those patients who were sensitive to first-line chemotherapy and chemotherapy before SCS. Cormio *et al* also restricted the subjects to patients with apparently isolated and resectable tumours and without ascites. Our criteria for patient selection were similar to those of Eisenkop *et al*, and their subjects were patients with DFI >6 months and without liver metastases. They achieved an 82% complete resection rate by using argon beam laser to remove disseminated cancer foci and reported 44 months in median survival and approximately 35% in 5-year survival in the completely resected cases. In our experience, median survival and 5-year survival in completely resected cases were 52 months and 47.6%, respectively, being much better than previous reports. Our rate of optimal cytoreduction, 84.1% (if defined as residual tumour <1 cm), was similar to the rate of complete resection in Eisenkop's report. In our series, optimally resected cases had 40 months in median survival and 38.6% in 5-year survival (figure not shown), in keeping with the survival of completely resected cases in Eisenkop's study. These findings suggest that the debulking efforts performed at SCS in our cases are comparable to those of previous reports.

Univariate analyses revealed that three factors during primary treatment (peritoneal spread, aortic lymph node metastasis, FIGO stage) and five factors at recurrence (DFI, liver metastasis, number of tumours, size of maximum tumour, SCS outcome) were significantly related to overall survival after recurrence. In the multivariate analysis excluding SCS outcome, the significance of all the three factors during primary treatment disappeared. Four factors determined at recurrence, that is, DFI, presence of liver metastasis, number of tumours and size of maximum tumour, were revealed to be independent prognostic factors.

DFI is the most important prognostic factor after recurrence, as described in many previous reports. In most studies, the cutoff period of DFI was set to 12 months. Two cutoff periods were set in Eisenkop's study (Eisenkop *et al*, 2000) (12 and 36 months) and in Tay's study (Tay *et al*, 2002) (12 and 24 months), and patients were divided into three groups. Although we also analysed our patients with DFI >12 months using cutoff periods such as 24 and 36 months, there were no significant differences between patients with and without DFI >24 or 36 months (data not shown). Recently, Zang *et al* (2004) performed SCS even in patients with DFI of 3 months and reported negative influence of DFI on overall survival. However, their follow-up period was only 16 months. This might be too short to detect a statistical difference.

Size of maximum tumour was also identified by Eisenkop *et al* (2000) as an independent prognostic factor. Eisenkop *et al* used 10 cm as the cutoff size, whereas we used 6 cm. The difference may be due to our earlier detection of recurrent tumours by using ultrasonography or CT scan within a 3-month interval. In our cases, there were only two patients in whom maximum tumour size exceeded 10 cm in diameter. At all events, tumour size seems to be an important factor reflecting biological aggressiveness of recurrent tumours.

The number of recurrent tumours has not been previously highlighted as a prognostic determinant. One reason is that some studies restricted the subjects for SCS to patients with isolated tumours or a solitary tumour (Cormio *et al*, 1999; Munkarah *et al*, 2001; Scarabelli *et al*, 2001). Another possible reason is that Eisenkop *et al* (2000) and Tay *et al* (2002) did not analyse the number of recurrent tumours as a factor influencing survival, although they pointed out that this factor may influence SCS outcome. In concordance with our results, Zang *et al* (2004) reported that the number of recurrent tumours influenced both overall survival and SCS outcome.

The current study revealed that liver metastasis is another important prognostic determinant. Vaccarello *et al* (1995) examined the relationship between site of recurrence and survival, and reported that liver metastasis had a negative influence on survival. In most studies, patients with liver metastasis were excluded from subjects for SCS. In our series, two patients with solitary liver metastasis were included: one patient underwent hepatic resection and the other patient did not undergo hepatic resection because of the presence of unresectable metastatic portal lymph nodes. They did not achieve good survival (20 and 14 months, respectively).

From the results of the multivariate analysis, we propose the following criteria for patient selection for SCS. Patients with recurrent ovarian cancer should be considered as ideal candidates for SCS when they have three or all of the following four factors at recurrence: (1) DFI >12 months, (2) no liver metastasis, (3) a solitary tumour and (4) tumour size <6 cm. Considering our original patient selection, we should propose exclusion criteria including (1) age at recurrence ⩾75 years, (2) PS 3 or 4 just before SCS and (3) progressive disease during presurgical chemotherapy, if undertaken. Although we used intraoperative findings for the number and size of tumours, size of maximum tumour was consistent between intraoperative findings and imaging in available cases. Therefore, we can accurately evaluate all these factors, except the number of tumours, before SCS. As for the number of tumours, ultrasonography or CT scan before SCS cannot always identify multiple peritoneal disseminated tumours. When the patient meets the criteria for SCS preoperatively, it is recommended to decide whether SCS should be accomplished after reconfirming the criteria at the time of laparotomy.

In the previous studies, several prognostic factors were shown to have significant correlation with overall survival of the patients. However, these factors were obtained from SCS in selected patients in most of the previous studies. In addition, how to use several significant prognostic factors to select good candidates for SCS was not fully analysed. To our knowledge, generally accepted or recommended selection criteria are ‘patients with longer DFI’ (Bristow *et al*, 1996; Roberts, 1996; Rose, 2000; Sijmons and Heintz, 2000). Thus, it was sometimes difficult to decide whether or not SCS should be performed in patients who have some favourable factors and a few unfavourable factors. We believe that our selection criteria for SCS should be helpful in deciding whether SCS should be performed.

In conclusion, our data suggest that patients with three or all four of the above-mentioned favourable factors are ideal candidates for SCS, and that the final decision should be made at laparotomy in borderline cases. It seems that SCS has a large impact on survival of patients with recurrent ovarian cancer when the patients are selected by the new criteria (47 months in median survival and 45.9% in 5-year survival). However, these patients were likely to have good sensitivity to chemotherapy, because they had DFI >6 months. In a recent trial of recurrent ovarian cancer with DFI >6 months, patients who received platinum-based chemotherapy with or without paclitaxel had a favourable prognosis: 29 and 24 months in median survival and around 20% in 5-year survival, respectively (Parmar *et al*, 2003). Although patients undergoing SCS using the new criteria of patient selection seem to have much better survival than patients receiving chemotherapy alone, our study was retrospective and noncomparative, and our data were based on a relatively small number of strictly selected patients. To provide solid evidence for the therapeutic benefit of SCS and to find better selection criteria for the surgery, further studies including randomised controlled studies are required.

## Figures and Tables

**Table 1:** Univariate analyses for variables during primary treatment

**Variables**	**Number**	**Median survival (months)**	***P*-value**
*Peritoneal tumour spread*			
Localised to the pelvis	10	NA	
Extended beyond the pelvis	34	29	0.039
			
*Stage*			
I/II	5	NA	
III/IV	39	29	0.045
			
*Aortic lymph node metastases*			
Absent	25	64	
Present	14	27	
Not assessed	5	25	0.009
			
*Pelvic lymph node metastases*			
Absent	20	47	
Present	21	32	
Not assessed	3	25	0.126
			
*Systematic lymphadenectomy*			
Not performed	3	25	
Pelvic only	7	29	
Pelvic and aortic	34	33	0.296
			
*Histology*			
Serous	35	37	
Others	9	23	0.197
			
*Residual tumour at PCS*			
0	34	32	
Any	10	40	0.961

PCS=primary cytoreductive surgery; NA=not applicable.

**Table 2:** Univariate analyses for variables at recurrence

**Variables**	**Number**	**Median survival (months)**	***P*-value**
*Age at recurrence (years)*			
<50	17	29	
⩾50	27	40	0.860
			
*Disease-free interval (months)*			
⩾12	31	47	
<12	13	23	0.002
			
*Intraperitoneal tumour*			
Absent	12	64	
Present	32	27	0.117
			
*Pelvic or aortic lymph node metastases*			
Absent	34	32	
Present	10	37	0.419
			
*Distant metastasis*			
Absent	38	32	
Present	6	40	0.496
			
*Liver metastasis*			
Absent	42	33	
Present	2	20	0.005
			
*No. of recurrent tumours*			
Solitary	16	64	
Multiple	28	27	0.007
			
*Size of maximum tumour (cm)*			
<6	38	40	
⩾6	6	14	<0.001
			
*Massive ascites (>500 ml)*			
Absent	41	33	
Present	3	32	0.318
			
*PS*			
0–2	42	29	
3	2	42	0.746
			
*Presurgical chemotherapy*			
Not done	23	33	
Done	21	29	0.677
			
*Bowel resection*			
Not done	33	33	
Done	11	27	0.650
			
*Residual tumour at SCS*			
0	26	52	
Any	18	22	0.002

PS=performance status; SCS=secondary cytoreductive surgery.

**Table 3:** Multivariate analysis using the seven prognostic variables in the univariate analyses

	**Multivariate analysis**	
**Variables**	**Risk ratio (95% CI)**	***P*-value**
*Peritoneal tumour spread at PCS*		
Localised to the pelvis	1.00	
Extended beyond the pelvis	0.80 (0.42–1.76)	0.540
		
*Stage*		
I/II	1.00	
III/IV	0.90 (0.22–5.60)	0.893
		
*Aortic lymph node metastases at PCS*		
Absent	1.00	
Present	1.23 (0.56–2.64)	
Not assessed	1.78 (0.61–5.33)	0.088
		
*Disease-free interval (months)*		
⩾12	1.00	
<12	2.45 (1.11–5.39)	0.027
		
*Liver metastasis*		
Absent	1.00	
Present	4.00 (1.40–10.03)	0.013
		
*No. of recurrent tumours*		
Solitary	1.00	
Multiple	3.73 (1.79–9.58)	<0.001
		
*Size of maximum tumour (cm)*		
<6	1.00	
⩾6	7.43 (3.12–18.92)	<0.001

PCS=primary cytoreductive surgery.
